# The Utility of Noninvasive Urinary Biomarkers for the Evaluation of Vesicoureteral Reflux in Children

**DOI:** 10.3390/ijms242417579

**Published:** 2023-12-17

**Authors:** Marius-Cosmin Colceriu, Paul Luchian Aldea, Andreea-Liana Boț (Răchişan), Bogdan Bulată, Dan Delean, Alina Grama, Alexandra Mititelu, Roxana Maria Decea, Alexandra Sevastre-Berghian, Simona Clichici, Tudor Lucian Pop, Teodora Mocan

**Affiliations:** 1 Discipline of Physiology, Department of Functional Biosciences, “Iuliu Hațieganu” University of Medicine and Pharmacy, 400006 Cluj-Napoca, Romania; roxana.decea@elearn.umfcluj.ro (R.M.D.); berghian.alexandra@umfcluj.ro (A.S.-B.); sclichici@umfcluj.ro (S.C.); teodora.mocan@umfcluj.ro (T.M.); 2 Second Pediatric Discipline, Department of Mother and Child, “Iuliu Haţieganu” University of Medicine and Pharmacy, 400177 Cluj-Napoca, Romaniaalinagrama1607@gmail.com (A.G.); perta.alexandra1@gmail.com (A.M.); tudor.pop@umfcluj.ro (T.L.P.); 3 Discipline of Public Health and Management, Department of Community Medicine, “Iuliu Haţieganu” University of Medicine and Pharmacy, 400006 Cluj-Napoca, Romania; paul.luch.aldea@elearn.umfcluj.ro; 4 Pediatric Nephrology, Dialysis and Toxicology Clinic, Emergency Clinical Hospital for Children, 400177 Cluj-Napoca, Romania; iggbie@gmail.com (B.B.);; 5 Second Pediatric Clinic, Emergency Clinical Hospital for Children, 400177 Cluj-Napoca, Romania; 6 Nanomedicine Department, Regional Institute of Gastroenterology and Hepatology, 400158 Cluj-Napoca, Romania

**Keywords:** vesicoureteral reflux, renal scars, reflux nephropathy, interleukin-6, cathelicidin, neutrophil gelatinase-associated lipocalin, children

## Abstract

Vesicoureteral reflux (VUR) is one of the most important disorders encountered in pediatric nephrology due to its frequency and potential evolution to chronic kidney disease (CKD). The aim of our study was to identify noninvasive and easy-to-determine urinary markers to facilitate the diagnosis and staging of VUR. We performed a cross-section study including 39 patients with VUR followed over three years (August 2021–September 2023) and 39 children without urinary disorder (the control group). We measured the urinary concentration of interleukin-6 (IL-6), cathelicidin (LL-37), and neutrophil gelatinase-associated lipocalin (NGAL) in VUR and healthy controls. Moreover, we analyzed the correlation between these biomarkers and the presence of renal scars (RS), reflux nephropathy (RN), and CKD. The NGAL concentrations were significantly higher in patients with VUR than in the controls (*p* = 0.02). Regarding the severity of the reflux, NGAL/creatinine and LL-37/creatinine were positively correlated with severe reflux (*p* = 0.04, respectively, *p* = 0.02). In patients with VUR and RS, LL-37/creatinine was significantly lower (*p* = 0.01). LL-37/creatinine with an AUC of 0.71 and NGAL/creatinine with an AUC of 0.72 could be acceptable diagnostic tests for severe VUR. In conclusion, urinary IL-6, NGAL, and LL-37 could serve as valuable markers for diagnosing and predicting outcomes in patients with VUR and RN.

## 1. Introduction

Vesicoureteral reflux (VUR) is the most common urinary tract anomaly, affecting 1% to 2% of all children, and is one of the main causes of hospitalization in pediatric nephrology departments [1]. VUR consists of an abnormal backward flow of urine from the bladder up to the ureter and urinary collecting system of the kidneys. It is determined either by a primary structural or functional abnormality of the vesicoureteral junction or secondary to a pathological condition that causes an increase in intravesical pressure, such as neurogenic bladder, posterior urethral valves, or other obstructive uropathies [2,3,4]. It is estimated that approximately one-third of patients with VUR will develop at least one episode of febrile urinary tract infection (UTI). On the other hand, up to 31% of children with febrile UTI can be diagnosed with VUR if they are appropriately investigated [5,6,7]. Due to recurrent pyelonephritis or the sterile pressure effect of urine on the renal structures, VUR can evolve with several long-term complications, like renal scars (RS), proteinuria, increased blood pressure, growth impairment, and chronic kidney disease (CKD). In 20–30% of cases, patients with VUR will develop RS. Reflux nephropathy (RN), defined as the presence of RS in association with VUR, can evolve into end-stage kidney disease (ESKD) in up to 25% of cases. VUR represents the etiology in 8.4% of CKD cases and 6% of ESKD cases among children and young adults [8,9,10].

Considering the epidemiological data mentioned above, with the secondary socioeconomic implications of repetitive hospitalizations, early diagnosis and proper management of VUR are mandatory. In order to facilitate the diagnosis, staging, long-term follow-up, and prevention of complications, new biomarkers related to the pathophysiology of VUR and RN are being studied.

Several studies conducted so far indicate urinary IL-6 as a potential biomarker in the diagnosis of VUR and RN, but the results do not allow drawing categorical conclusions. IL-6 is a proinflammatory cytokine involved in the immune response during UTIs and in the inflammatory processes that occur in the renal parenchyma, with possible involvement in the pathophysiology of RN. Most of the studies reported the presence of increased concentrations of urinary IL-6 in patients with VUR or RS. The results published up to this moment encourage further studies [11,12,13].

Cathelicidin (LL-37) is an antimicrobial peptide (AMP) with a role in the early stages of the innate immune response during UTIs. Gupta et al. have shown that urinary concentrations of LL-37 are higher in patients with obstructive urologic disorders. Thus, considering the involvement of IL-37 in the pathogenesis of recurrent UTI and possible involvement in other urological pathologies, such as obstructive uropathy, we consider LL-37 a possible biomarker for RN [14,15,16,17].

Another possible urinary biomarker for renal disorder studied recently is neutrophil gelatinase-associated lipocalin (NGAL). It is a protein that binds siderophores, thus interfering with the iron metabolism necessary for bacterial survival and replication. In the kidneys, NGAL is secreted in the proximal tubular epithelial cells in the early stages of UTIs, after ischemia, and during the recovery process after renal injury. Some of the studies conducted so far revealed the value of urinary NGAL in diagnosing VUR, but these results are not unanimously supported. Regarding RS detection, the studies suggest that higher concentrations of urinary NGAL were found in patients with VUR and RS compared with those with VUR without RS. Thus, NGAL may be a predictive biomarker for RN and its progression to ESKD [18,19,20,21].

The aim of our study was to identify noninvasive and easy-to-determine urinary biomarkers (IL-6, NGAL, and LL-37) that could be used in the diagnosis and staging of VUR and RN. In addition, we evaluated the correlation between these biomarkers and the presence and severity of RS, RN, and CKD. Moreover, we aimed to add new data to the current level of knowledge regarding the physiopathology of RN to improve the clinical approach for these patients. 

## 2. Results

### 2.1. Patient Characteristics

Urine specimens were obtained from 78 children (aged between 1 month and 17 years), of which 39 (20 girls, 19 boys) were diagnosed with VUR and 39 (16 girls and 23 boys) were healthy controls. There were no significant differences in age (*p* = 0.62), sex, and nutritional status (*p* = 0.87) between groups. In the patient’s group, 17 (43.6%) children had unilateral VUR and 22 (56.4%) had bilateral VUR. Regarding the severity of VUR in the studied group, 11 (28.2%) patients had mild VUR, 18 (46.2%) had moderate VUR, and 10 (25.6%) had severe VUR. Nine (23%) patients presented RS on DMSA scan, and seven (17.9%) presented impaired renal function based on eGFR. No significant correlations existed between the numbers of UTIs and RS or RN in our cohort. Also, we did not find significant correlations between the three severity groups of VUR and RS or the number of UTI episodes (*p* = 0.09). 

### 2.2. Urinary IL-6, NGAL, and LL-37 Concentrations

**Figure 1:**
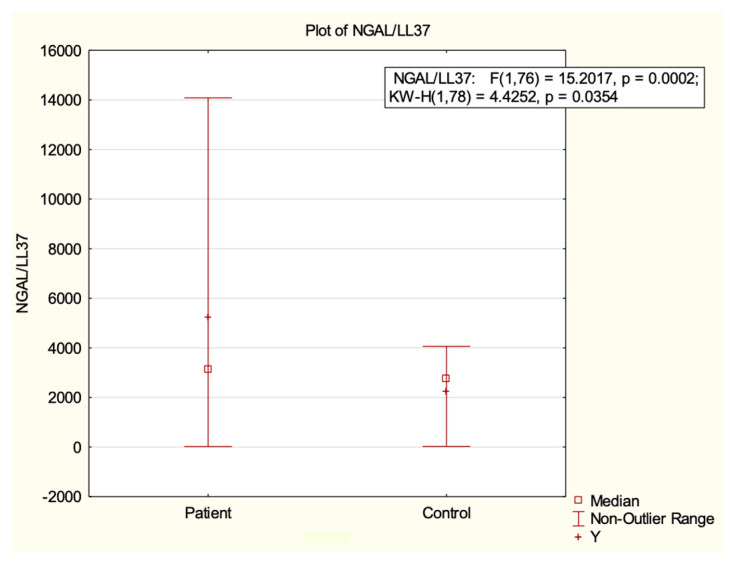
A significant difference in the NGAL/LL-37 ratio was observed between patients with VUR and the controls.

The urinary levels of IL-6, NGAL, LL-37, and microalbuminuria (μAlb) are shown in Table 1. Urinary NGAL levels were significantly higher in patients with VUR than controls (*p* = 0.02). Comparing patients with controls, there were no significant differences between urinary median levels of IL-6 (1.83 pg/mL vs. 1.75 pg/mL) and LL-37 (0.85 ng/mL vs. 0.84 ng/mL). In order to standardize the samples, we reported the results as ratios based on urinary creatinine levels. The NGAL/creatinine ratio was significantly higher in patients (*p* = 0.03). Also, the NGAL/LL-37 ratio significantly differed between groups (*p* = 0.03; Figure 1). The results for IL-6/creatinine, LL-37/creatinine, and μAlb/creatinine were higher in patients, but without statistical significance (*p* = 0.22, *p* = 0.65, and *p* = 0.73, respectively).

**Figure 2:**
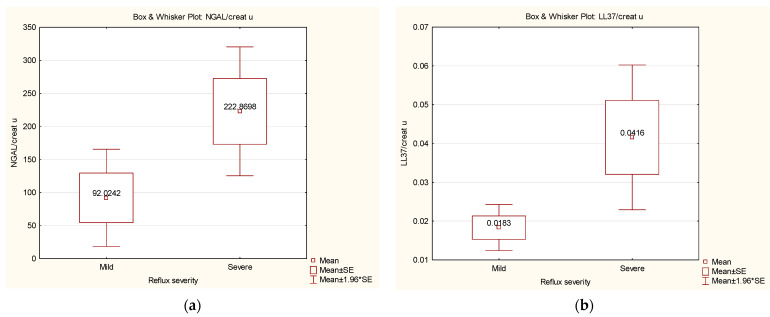
(**a**) A significant difference in the NGAL/creatinine ratio was observed between patients with mild and severe VUR. (**b**) A significant difference in the LL-37/creatinine ratio was observed between patients with mild and severe VUR.

**Figure 3:**
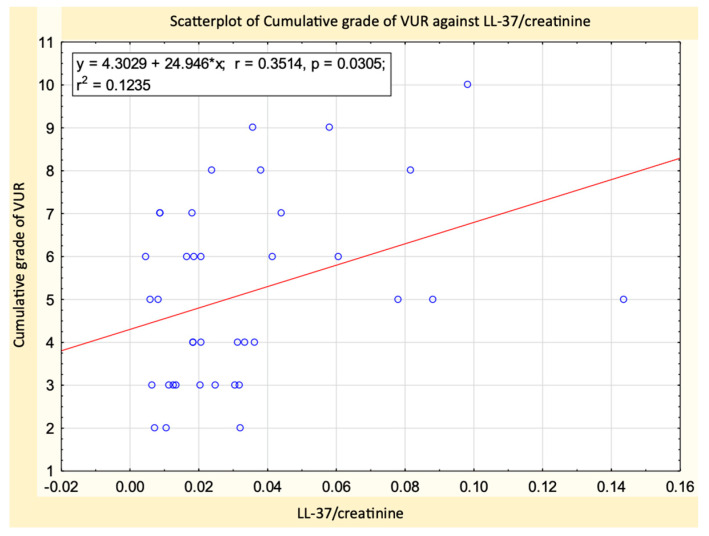
As the degree of VUR increases, there is a corresponding increase in the LL-37/creatinine ratio.

Regarding the severity of VUR, our results show higher values for NGAL/creatinine (Figure 2a) and LL-37/creatinine (Figure 2b) in patients with severe VUR compared with those with mild VUR (*p* = 0.04 and *p* = 0.02, respectively). There were also higher values for μAlb/creatinine, IL-6/creatinine, and NGAL/LL-37, but without statistical significance. No significant differences were found between patients with mild and moderate VUR, or between those with moderate and severe VUR. We also observed a statistically significant (*p* = 0.03, R = 0.3) relationship of direct proportionality between LL-37/creatinine and the cumulative grade of VUR (Figure 3). 

### 2.3. Results Regarding RS, RN, and CKD

In patients with VUR and RS, urinary creatinine levels were significantly higher compared to patients with VUR without RS (16.7 mg/dL vs. 28.6 mg/dL, *p* = 0.004). Within the same comparison, the levels of LL-37/creatinine were lower in patients with VUR and RS (*p* = 0.01). Levels of IL-6 and NGAL were higher in patients with RS (23.9 pg/mL vs. 18.1 pg/mL, and 21.5 pg/mL vs. 18.8 pg/mL, respectively), but without statistical significance. IL-6/creatinine, NGAL/creatinine, and μAlb/creatinine did not present significant differences between the two groups. Our study found no correlations between the noninvasive markers studied and RN, high blood pressure, the numbers of UTIs, or CKD (stages 2–5, defined as eGFR <90 mL/min/1.73 m^2^). 

### 2.4. ROC Curve Analysis

**Figure 4:**
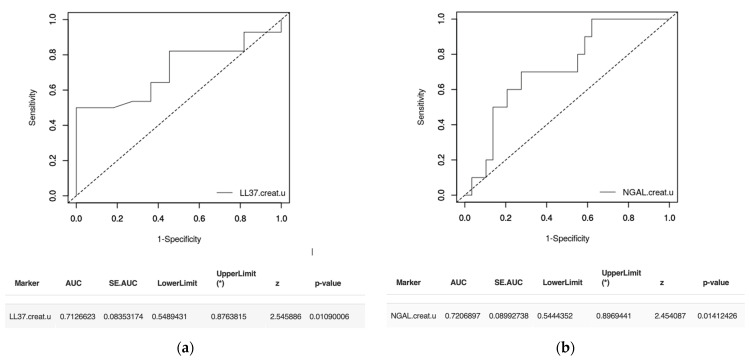
(**a**) ROC curve showing the LL-37/creatinine value in the diagnosis of severe VUR. (**b**) ROC curve showing the NGAL/creatinine value in the diagnosis of severe VUR.

An ROC curve was used to determine the predictive value of NGAL, NGAL/creatinine, and NGAL/LL-37 in diagnosing VUR. The ROC curve drawn for NGAL (*p* = 0.01) revealed an AUC of 0.65, while the one for NGAL/creatinine (*p* = 0.02) revealed an AUC of 0.64. Both represent poor diagnosis tests for VUR. With the same diagnostic significance, the ROC curve for NGAL/LL-37 (*p* = 0.03) presented an AUC of 0.64. For predicting severe VUR, we analyzed the ROC curves for NGAL/creatinine, NGAL/LL-37, and LL-37/creatinine. The results showed that LL-37/creatinine, with an AUC of 0.71 (Figure 4a), and NGAL/creatinine, with an AUC of 0.72 (Figure 4b), could be acceptable diagnostic tests for severe VUR. The curve for NGAL/LL-37 revealed an AUC of 0.67, with a poor predictive value. 

## 3. Discussion

Our study starts from the premise that certain effectors of innate immunity involved in the physiopathology of RN could be used as noninvasive diagnostic and prognostic markers. Thus, we tried to determine the role of urinary IL-6, NGAL, and LL-37 as potential biomarkers in diagnosing VUR, RS, and RN. Moreover, we tried to establish their correlations with VUR severity, the number of UTI episodes, and CKD. 

IL-6 is a small protein involved in the immune reaction during UTIs. It exerts a proinflammatory activity, having an important role in the acute-phase reaction, including the development of fever. In the kidneys, IL-6 is secreted by tubular, mesangial, and endothelial cells, and is involved in local inflammatory processes [22,23]. Immunohistochemical assessments performed in patients with RN have shown that areas of increased IL-6 synthesis are located near areas of fibrosis [24]. These aspects support the possible involvement of IL-6 in the pathophysiology of VUR and RN. 

Our study revealed that urinary IL-6 and IL-6/creatinine were higher in patients with VUR than in healthy children. However, the differences were not statistically significant. We also obtained higher levels of urinary IL-6 in patients with RN and RS than the control, but still without statistical significance. Regarding the discrimination of reflux severity, we did not find differences in the urinary IL-6 and IL-6/creatinine between the groups.

The results of similar studies are presented in Table 2. 

No differences in urinary IL-6 levels between patients with and without VUR were reported in some studies [25,26,27,28,29], but others supported the diagnostic potential of urinary IL-6 for VUR [11,12,13]. Most of the studies [24,25,28,29] revealed higher urinary IL-6 levels in patients with RS compared with controls, but others did not find any significant difference [12,13,30]. 

**Table 2:** Previous studies regarding IL-6 in VUR.

Authors	Year	Description	Results
Ninan et al. [25]	1999	32 children: 11 with VUR and RN, 6 with VUR, and 15 controls	No differences between patients with VUR and the controls. A significant increase in IL-6 levels was observed in patients with RN.
Haraoka et al. [26]	1996	32 children with VUR without UTI	No differences between patients with VUR and controls. Urinary IL-6 levels were below the lower limit of detection (10 pg/mL).
Cordoba et al. [27]	2012	Case-control study on 40 patients who underwent a VCUG	No differences between patients with VUR and controls.
Grupta et al. [30]	2020	23 patients with a history of febrile UTI and RS and 27 patients with a history of febrile UTI without RS	No differences in urinary IL-6 levels between the two groups.
Sheu et al. [28]	2009	45 children (aged between 1 and 120 months) with acute pyelonephritis and 34 with lower UTI	No differences between patients with VUR and controls. Elevations in urinary IL-6 levels were correlated with an increased risk of subsequent RS.
Tramma et al. [29]	2012	23 children with VUR and RS, 10 children with RS without VUR, 4 children with VUR without RS, and 13 controls	No differences between patients with VUR and controls. Levels of urinary IL-6 were correlated with the severity of RS.
Wang et al. [24]	2001	66 children with RS	Strong correlation between RS and urinary IL-6 levels.
Nickavar et al. [13]	2020	70 children (34 with VUR, 36 controls)	Significantly higher urinary IL-6 levels in patients with VUR. No correlation between urinary IL-6 and the severity of VUR or RS.
Gokce et al. [12]	2010	114 children (26 with VUR and RS, 27 with VUR without RS, 34 with RS without VUR, and 27 controls)	Significantly higher urinary IL-6 levels in patients with VUR (poor diagnostic test—AUC of 0.64). Weak correlation between IL-6 and the grade of the VUR. No differences between patients with RS and controls.
Krzemien et al. [11]	2004	33 children aged 1–24 months with first-time UTI	Higher urinary IL-6 concentrations in patients with VUR.

VUR—vesicoureteral reflux; RN—reflux nephropathy; IL-6—interleukin-6; UTI—urinary tract infection; VCUG—voiding cystourethrogram; RS—renal scar; AUC—area under the ROC curve; ROC—receiver operating characteristic.

The variability of published results may be explained by the relatively small number of patients in the case of most studies. Another possible explanation for the differing results could be the variations in the lower detection limits of urinary IL-6 and differences in sample analysis procedures. However, despite the differences between the results obtained, the authors of these publications support the possibility of using IL-6 as a diagnostic and prognostic urinary marker in VUR. This is based on scientific evidence of the involvement of IL-6 in the processes of inflammation and fibrosis, which lead to RS formation secondary to VUR. These processes continue in the absence of UTI, implying an increase in urinary IL-6 concentrations apart from UTIs. There are also patients with RS secondary to VUR without UTIs. In these cases, infiltration with lymphocytes, monocytes, and other immune cells in the tubular interstitium was observed. This infiltrate is responsible for IL-6 and other IL secretion [12]. Another pathophysiological aspect that supports the increased expression of urinary cytokines in patients with VUR and RN is linked to Toll-like receptors (TLRs). TLRs are involved in the innate immune response during UTIs, resulting in proinflammatory cytokine secretion, such as IL-6. TLR4 is upregulated in the kidneys after parenchymal injury. Mice deficient in TLR4 are protected against histological damage and renal dysfunction due to reducing the proinflammatory cytokine secretion [31,32]. Patients with RS secondary to recurrent UTIs may have upregulated TLR4 expression, resulting in chronic cytokine secretion [29]. Further studies are needed to exploit these physiopathological changes in clinical practice. 

NGAL is a peptide highly secreted in urine during UTI, as it has antimicrobial activity. Secondary to the increased production and decreased reabsorption, urinary NGAL concentrations also increase in renal parenchymal damage. During RS formation, the increase in the urinary concentration of NGAL is attributed to the regeneration of renal tubular cells. These aspects propose NGAL as a potential urinary marker with possible usefulness in managing patients with VUR [33,34].

Our study revealed significant differences in urinary NGAL concentrations and NGAL/creatinine ratios between patients with VUR and controls. We also found that patients with VUR have an increased NGAL/LL-37 ratio, with statistical significance compared to controls. Considering the increase in these three urinary parameters as a potential tool in VUR diagnosis, we resorted to ROC–AUC analysis. The ROC curve indicates a poor test value in diagnosing VUR for all three markers (an AUC between 0.6 and 0.7). NGAL/creatinine turned out to be useful in differentiating severe from mild VUR. This aspect could be useful in identifying patients with VUR that require surgical intervention. Even though urinary NGAL levels were higher in patients with RS, our results did not support its use as a diagnostic marker for RS. 

In their studies, Nickavar et al., Parmaksiz et al., and Ichino et al. showed that patients with VUR had significantly higher urinary NGAL concentrations than controls. The same studies also support the value of urinary NGAL as a diagnostic marker for RS [21,35,36]. Moreover, the study conducted by Eskandarifar et al. supports NGAL as an excellent diagnostic test with a high specificity for RS. They also found that urinary NGAL concentrations were significantly higher in patients with VUR than in controls [20]. In their studies, Naik et al. and Ganapathy et al. also support the value of urinary NGAL in the diagnosis of RS [18,37]. 

In all of the aforementioned studies, the diagnostic value of urinary NGAL in RS was emphasized. They also demonstrated that high urinary NGAL is associated with the presence of VUR, but none support the correlation between NGAL and the severity of VUR. In contrast, our study did not support NGAL as a reliable diagnostic marker for RS, but it showed its value in diagnosing severe VUR. The physiopathology of RS development could explain this aspect. RS may appear secondary to VUR and recurrent UTIs, but also in the presence of VUR without UTIs. In this latter situation, the alteration of the renal parenchyma occurs due to the sterile pressure effect of the refluxed urine. Patients present small kidneys in these cases, while RS in patients with recurrent UTIs appear as defects on the DMSA scan [36,38]. Ichino et al. found in their study that small kidneys have a lower capacity to synthesize NGAL [21,36]. In our study, approximately 25% of patients had small kidneys, which could explain the differences between the results. Further studies should evaluate urinary markers separately for the two mechanisms of RS development due to potential differences in cytokines and AMP expression.

Cathelicidin (LL-37) is an AMP synthesized by monocytes, neutrophils, natural killer (NK) lymphocytes, myeloid bone marrow cells, and epithelial cells. In sterile urine, the concentration of LL-37 is very low. Immediately after bacterial colonization, the urothelium secretes LL-37 as part of the innate immune response. Considering the involvement of recurrent UTIs in the pathophysiology of RN, LL-37 could be a potential marker for VUR and RN [17,39].

To the best of our knowledge, there is no study conducted so far regarding LL-37 involvement in VUR. Gupta et al. previously studied LL-37 as a potential marker in obstructive uropathies. Their results revealed a good prediction value of LL-37 in diagnosing obstructive uropathies [17].

In our study, urinary levels of LL-37 and LL-37/creatinine were higher in patients with VUR than the controls, but the difference was not statistically significant. We observed a significantly higher NGAL/LL-37 ratio in patients with VUR, but its diagnostic value was poor. The LL-37/creatinine ratio correlated with the grade of VUR (expressed as a cumulative scale from 1 to 10) and had an acceptable prediction value for severe VUR. We also found that LL-37/creatinine was significantly lower in the patients with RS. 

Thus, it could be a reliable marker to predict RS occurrence in patients with VUR. Patients with high-grade VUR may have high levels of urinary LL-37 due to the innate immune reaction and proinflammatory state during RS development. On the other hand, the low levels of urinary LL-37 associated with RS may be explained by the destruction of tubular epithelial cells, which is the site of LL-37 renal production. To confirm this, immunohistological studies are required. 

The main limitation of our study was the small number of children with VUR. In addition, as mentioned before, the high number of patients with small kidneys enrolled in the study could also have influenced the results. To establish the clinical significance and include these urinary markers in the guidelines for the medical management of VUR, further studies with larger age- and sex-matched groups are needed. Standardizing sample handling and strict inclusion/exclusion criteria can improve research results.

Our results encourage further studies to evaluate LL-37, IL-6, and NGAL as noninvasive urinary markers that can improve the management of patients with VUR. Moreover, these urinary biomarkers may become alternative assessment tools to VCUG and DMSA scans. According to our study, urinary NGAL and LL-37 can be useful in differentiating severe from mild VUR. Thus, it could be easier to identify patients who require antibiotic prophylaxis or surgical intervention. All these aspects would increase patient comfort and reduce the costs related to hospitalizations and investigations. Also, due to the prognostic value of these markers in diagnosing RS and RN, it may be easier to identify patients at risk of developing CKD, allowing prompt therapeutic intervention that would improve clinical outcomes. 

## 4. Materials and Methods

### 4.1. Patient Enrolment

We enrolled 39 children diagnosed with VUR in this cross-sectional study who were hospitalized in the Department of Pediatric Nephrology, Dialysis and Toxicology of the Emergency Clinical Hospital for Children, Cluj-Napoca, Romania, over a period of three years (August 2021–September 2023), alongside 39 children without urinary illness (the control group). All the children were free of UTI for at least one month before urine sample collection. Individuals with UTIs or other inflammatory illnesses at the moment of the study were excluded. Thus, before inclusion in the study, any inflammatory or infectious disease was ruled out by clinical examination, white blood cell count (WBC), C-reactive protein (CRP), the erythrocyte sedimentation rate (ESR), and urine analysis (dipstick, sediment, and culture). 

This study was carried out based on approval issued by the Ethics Committee of the Emergency Clinical Hospital for Children in Cluj-Napoca, Romania (no.168/28.05.2021), after receiving informed consent from the parent/guardian.

### 4.2. Study Procedures

We analyzed the demographic characteristics of the patients (age, gender, nutritional status according to CDC criteria), clinical parameters, comorbidities, familial history for VUR, number of UTIs in antecedents, VUR degree, laterality, presence of RN, and CKD.

Urine samples were collected at the first morning voids. In children with full bladder control, we obtained midstream urine samples. For children without adequate bladder control, we used urine collection bags. Immediately after collection, we performed a urinalysis and urine culture. Also, we determined urinary creatinine levels, microalbuminuria, and urine albumin-to-creatinine ratio (UACR). At the same time, we determined WBC, ESR, CRP, blood urea nitrogen, serum creatinine, and the estimated glomerular filtration rate (eGFR). Following that, urine specimens were centrifugated at 3500 revolutions per minute for 5 min and stored at −20 °C. Samples stored for more than one month were kept at −80 °C. 

For the diagnosis of VUR, we used the medical records and voiding cystourethrogram (VCUG). The VCUG is the gold standard investigation for the diagnosis of VUR and consists of bladder catheterization with the inoculation of a contrast agent, followed by a radiological examination. The VCUG allows the diagnosis of VUR, staging, and description of the anatomy of the urinary tract, and is also indicated for tracking the evolution of VUR [7,40].

The grading of VUR was established according to the International Reflux Classification outlined by the International Reflux Study Group using a scale from I to V [41,42]. When bilateral VUR was present, we used a scale of 1 to 10 to cumulate the degree of VUR. Subsequently, we grouped the patients with VUR in 3 degrees of severity: mild (1–3), moderate (4–6), and severe (7–10). 

DMSA (99 m Tc-dimercaptosuccinic acid) scintigraphy was performed in patients with VUR to evaluate RS and RN. Renal cortical defect, small kidney, and impaired renal function were considered suggestive aspects for the diagnosis of RN on DMSA examination. The scintigraphic investigation was performed at least 3 months after acute pyelonephritis [43].

Acute febrile UTI was defined as acute onset of fever (greater than 38 °C), pyuria (white blood cell count more than 10 per high power field in urine sample sediment), positive urine bacterial culture with >100,000 colony-forming units/mL (CFU/mL), CRP greater than 2 mg/mL, procalcitonin level greater than 1, and WBC of 12,000/mL or greater [44].

Based on eGFR, patients with VUR were diagnosed with the corresponding stage of CKD (1 to 5). Here, eGFR was calculated using the Schwartz pediatric bedside formula for patients up to 16 years old (0.413 × height/serum creatinine). For those between 16 and 18 years old, we used the CKD–EPI equation based on serum creatinine (online tool) [45,46].

### 4.3. Urinary Markers Assay

Urinary IL-6, NGAL, and LL-37 were measured. Prior to analysis, the urine samples were thawed at room temperature. The LL-37 level was analyzed using a Human LL-37 (antibacterial protein LL-37) ELISA Kit, Elabscience, Houston, TX, USA. For urinary NGAL analyses, we used the human NGAL (neutrophil gelatinase-associated lipocalin) ELISA Kit, Elabscience, Houston, TX, USA. For urinary IL-6, we used a human IL-6 ELISA Kit, Elabscience, Houston, TX, USA. These ELISA kits use the sandwich ELISA principle. The ELISA microplates were precoated with antibodies specific to IL-6, HGAL, and LL-37. All the determinations were performed following the manufacturer’s recommendations and the instructions stated in the user manuals. The results were read on an automated microplate reader (Dynex DS2, Dynex Technologies Inc., Chantilly, VA, USA). The lower detection limit was 0.095 pg/mL for IL-6, 0.129 ng/mL for LL-37, and 0.116 pg/mL for NGAL. In order to standardize samples, avoid dilution effects, and obtain comparable results, the measured values were expressed as ratios to urinary creatinine. The latter was measured by spectrophotometry using the Mindray SAL 6000 analyzer (Mindray Bio-Chemical Electronics Co., Ltd., Shenzhen, China). 

### 4.4. Data Analysis and Statistics

All obtained data were included in a database set up in Microsoft Office Excel and statistically interpreted using the Statistica software, Version 13, TIBCO Software Inc., Palo Alto, CA, USA. We used descriptive statistics for continuous distribution variables (median and ranges), and statistical relevance was tested using the Mann–Whitney U test. We calculated the Spearman’s rank correlation coefficient and drew a graphic representation of the regression line. The associations between qualitative and continuous numeric variables were analyzed using the variant analysis method (the ANOVA test). The predictive accuracy for the prognostic role of urinary IL-6, NGAL, and LL-37 was assessed using receiver operating characteristic (ROC) curves, area under curve (AUC) values, confidence intervals, and cut-off values. An AUC of 0.5 was of no value, 0.51–0.69 was a poor test, 0.7–0.79 was considered acceptable, 0.8–0.89 was excellent, greater than 0.9 was outstanding, and 1 was a perfect test [47]. In all analyses, the results were considered significant at *p* < 0.05. For *p* values <0.01, we considered the test to have a good statistical significance, while *p* < 0.001 indicated that the statistical significance was extremely important (with an error margin of 0.1%).

## 5. Conclusions

Urinary IL-6, NGAL, and LL-37 could serve as valuable markers for diagnosing and predicting outcomes in patients with VUR and RN. These biomarkers could help to identify the severity of kidney injury in children with VUR. Based on our results and similar ones previously published, future prospective studies will be able to establish the role of these markers in the early detection of patients at risk for unfavorable evolution. Moreover, these noninvasive and easy-to-determine urinary markers can facilitate the monitoring of evolution, replacing invasive and laborious examinations, such as the VCUG. Besides the diagnostic and prognostic potential, the markers studied by us can influence the clinical management of patients with VUR. They could be used to distinguish patients who require surgical intervention from those who can be managed with prophylactic antibiotic therapy or those who can be only followed up clinically. However, further research is needed for their implementation in clinical practice.

## Figures and Tables

**Table 1:** Comparison between children with VUR and controls.

Characteristics	Patients (Median)	Controls (Median)	Patients Range	Controls Range	*p* Value
Age (years)	3.91	5.83	0.33–16.33	0.04–17.00	0.62
W/H Z score	0.07	0.15	−3.12–2.18	−7.30–2.00	0.87
eGFR (ml/min/1.73 m^2^)	109	117	42.00–170.00	90.00–179.00	0.07
Serum creatinine (mg/dL)	0.37	0.37	0.12–1.18	0.19–0.70	0.70
Urinary creatinine (mg/dL)	40.19	43.95	1.13–172.92	2.65–254.62	0.99
uACR	0.01	0.008	0.002–1.40	0.004–0.47	0.73
IL-6 (pg/mL)	1.83	1.75	1.33–135.10	1.33–3.15	0.14
IL-6/creatinine (pg/mg)	0.06	0.05	0.01–16.89	0.009–0.63	0.22
LL-37 (ng/mL)	0.85	0.84	0.71–3.03	0.68–1.11	0.21
LL-37/creatinine (ng/mg)	0.02	0.01	0.005–0.79	0.003–0.33	0.65
NGAL (pg/mL)	2755	2297	20.60–10,000	23.00–3115	0.02
NGAL/creatinine (pg/mg)	64.19	40.15	8.01–1075.26	0.76–963.77	0.05
NGAL/LL-37 (pg/ng)	3125	2744	23.15–14,084	29.11–4061.33	0.03
IL-6/LL-37 (pg/ng)	2.24	2.07	0.55–168.87	1.36–3.86	0.61

W—weight; H—height; eGFR—estimated glomerular filtration rate; uACR—microalbuminuria/creatinine ratio; IL-6—interleukin-6; LL-37—cathelicidin; NGAL—neutrophil gelatinase-associated lipocalin.

## Data Availability

Data is contained within the article.
